# Teachers Self-Efficacy and Employee Brand Based Equity: A Perspective of College Students

**DOI:** 10.3389/fpsyg.2022.884538

**Published:** 2022-04-26

**Authors:** Meiyang Li

**Affiliations:** Department of Architecture and Technology of Resources Engineering, Xi’an University of Architecture and Technology, Xi’an, China

**Keywords:** self-efficacy, brand equity, student engagement, employee based brand equity, teachers, students

## Abstract

Teachers working in institutions like to affiliate themselves with their organizations taking into account their efficacy toward jobs along with encouraging students in studies. The main objective of the present study is to identify the teachers’ self-efficacy on collective self-efficacy, academic psychological capital, and students’ engagement which consequently affect brand-based equity. The population taken in this study is college students across China, deriving a sample size of 316. The sample has been selected on the basis of the convenience sampling technique. Smart PLS 3.3.5 software has been used in the present study to analyze data for structural equation modeling. The findings of the study had shown that teachers’ self-efficacy does not have any impact on employee-based brand equity, however, significantly affects collective self-efficacy, academic psychological capital, and student engagement. Further, strong and moderate mediations have also been confirmed in the study for collective self-efficacy, academic psychological capital, student engagement between the relationships between teachers self-efficacy and brand based equity, however, collective self-efficacy and academic psychological capital could not find any mediating significance. The results have identified the significant role of teachers’ self-efficacy for collective self-efficacy, academic psychological capital, and student engagement. These empirical findings suggest policy implications for the retention of students in colleges across China.

## Introduction

Various studies link effective brand-creation by businesses to increased stakeholder value and competitive advantage (Wiles et al., 2012). The literature indicates that brand equity serves a strategic function in assisting management in creating a competitive edge. The resource-based perspective of the business theorizes that a business’s difficulty is to imitate capabilities that will allow it to build and retain superior advantages (Wernerfelt, 1984; Barney, 1991). College management is increasingly realizing that having a good knowledge of the brand equity notion may help them discover the characteristics that bring value to the brands and position them well in the market (Partenie, 2019; Dajani et al., 2021). As a result, college administration should have a better understanding of their brand equity in order to recruit students and faculty. Moreover, there is hardly any scientific publication on management comprehension of college brand equity, which is fascinating. In particular, when it comes to brand management scholarship in emerging markets, less is known about the notion (Pinar et al., 2014).

Multiple studies have been conducted on the branding and marketing of institutions of higher learning over the years. The focus of preliminary studies appears to be focused on marketing and advertising aspects (Argenti, 2000; Fazli-Salehi et al., 2019). Academic program repute, tuition, pamphlets, relationships between teachers, students, employees, and electronic media, as well as publicity, are all key variables for students when choosing a business school (Ryńca and Ziaeian, 2021). Other research works have focused on the role of interactions between teachers, students, staff, and the community in university marketing. Furthermore, amenities were discovered to be crucial for institution branding (Girard and Pinar, 2021). Conversely, there have also been concerns raised about the efficacy of such promotional operations, as well as the rising usage of similar catchphrases and slogans for university branding (Mingione et al., 2020). Whereas these investigations gave considerable information on the relevance of these characteristics for college branding and marketing but did not include evidence of how often institutions succeed in satisfying expectations on these factors. Furthermore, Hemsley-Brown et al. (2016) stated that research work exploring diverse sectors of schooling has expanded the knowledge of branding. Brand meaning, brand equity, the influence of educational brands on students, graduates, and staff, brand identity, and brand reputation were among the topics discussed (Naidoo et al., 2014; Balaji et al., 2016; Dean et al., 2016; Palmer et al., 2016; Plewa et al., 2016; Rauschnabel et al., 2016; Wilson and Elliot, 2016; Saurombe et al., 2017). According to Ng and Forbes (2009), the main value of university branding is the student learning experience.

Pinar et al. (2014) defined the fundamental value chain indicators that are necessary for building a solid college brand and brand equity relying on the marketing literature. Being part of the student college learning experience, the core value production activities are complemented by a supporting value chain (Ng and Forbes, 2009). Pinar et al. (2020) also highlighted key supportive actions for developing college branding. Both core and supportive elements are used in college branding in a manner that is compatible with and pertinent to the fundamental and supportive associations for service (Altaf et al., 2019). Functional areas, in fact, serve to distinguish and provide value to service brands (Altaf et al., 2019). Pinar et al. (2014) devised and verified a measurement scale for the main and supporting brand equity elements that are critical to the development of a successful university brand. Researchers did not give any data on how well these brand equity aspects performed in terms of meeting or failing to meet students’ expectations in creating a solid university brand (Girard and Pinar, 2021).

Teacher self-efficacy (TSE) has been extensively researched in connection to a variety of variables. Highly effective teachers are risk-takers who instill higher expectations in their classrooms, which leads to improved student performance (Burić and Moè, 2020; Todd, 2021). In a larger sense, teaching self-efficacy is said to be linked to other qualities such as quality of work-life, perfection, and interpersonal skills. Teacher effectiveness, on the other hand, has been shown to be adversely connected with teacher burnout. When all of the research are combined, it’s clear that there are still concerns with teachers’ self-efficacy that have to be examined further (Fathi and Saeedian, 2020; Lazarides et al., 2020; Saidi, 2020). The preponderance of research on teachers’ self-efficacy was quantifiable (Alibakhshi et al., 2020). Furthermore, as self-efficacy is situational and multidimensional, the objectivist instruments of investigation such as inferential or empirical research would not be suited for examining such an entity. As a result, the interpretive research paradigm and instruments may provide a more accurate representation of the notion (Alibakhshi et al., 2020).

Collective efficacy is an institution’s conviction in its people’s capacity to execute tasks effectively or a common understanding of a company’s capacity to attain specified goals. “Any collective conviction in the accomplishment of a certain endeavor, which represented the intention of a community to execute a particular goal” (Fan, 2018). There are four causes of parameters influencing (Bandura, 1982). Affective mastery (previous performance), vicarious experience, verbal persuasion, and physiological and emotive states are listed in the highest to the lowest order of significance. Collective effectiveness is formed by team members acquiring, storing, manipulating, and exchanging knowledge about one another, as well as their job, environment, processes, and prior performance. Collective efficacy has previously been studied as a mediator in the context of teachers’ self-efficacy (Fan, 2018) therefore, the current research also tried to assess its mediating role in developing employee-based brand equity at the college level.

Students will be joining competitive, extremely quick, globalized, and unpredictable jobs. Employers are constantly relying on their human resources to develop and maintain a competitive edge. These human resource management must contribute something beyond technological skills, academic talents, contacts, and networking (social capital) to the table; they must also contribute strong psychological capital to the table. As a result, if the main purpose of higher education is to equip our students to be successful in the workforce, we must move beyond teaching technical and intellectual skills to focus on the development of a person’s positive psychological capital (Luthans et al., 2013; Onivehu, 2020; Kang et al., 2021). In the current scope of our study, it was assumed that academic psychological capital could aid in the relationship between teachers’ self-efficacy and employee-based brand equity.

The term student engagement in class is a relatively recent term that has been characterized in a variety of ways by various scholars (Kahu and Nelson, 2017; Allen et al., 2018; Barkley, 2018). It can therefore be explained as a feeling of civic connectedness or participation, or as an experience depending on a variety of variables (Jain et al., 2013; Bear et al., 2019). Students are considered to be motivated to fully participate in their classrooms by a sense of engagement, which has been scientifically shown in a variety of circumstances and socioeconomic contexts. Effective social and academic outcomes are intimately linked to certain motivated engagement and suitability. This leads one to believe that perhaps the teacher’s efforts are reflected in the students’ emotions of participation (Siddiqi, 2018). Due to these logical connections, it was assumed that by playing a mediating role, student engagement could aid in developing employee-based brand equity.

Horan et al. (2011) claimed that, while brand equity is a developing component of the research, it has only been studied from neither the viewpoint of consumers nor from the perspective of management. Furthermore, a review of the literature indicates that studies in the service industry, particularly in the college environment, are still scarce (Pinar et al., 2020). As a result, this work is poised to fill a gap in the literature. Understanding the advantages of marketing, empirical insights into university brand management methods, variables influencing student choice of an institution, and brand equity in schooling are just a few of the publications available. No study evaluated the relationship of teachers’ self-efficacy in developing employee-based brand equity at higher educational institutes like colleges. Therefore, this study could drive a new dimension in the field of higher education employees based on brand equity.

## Theoretical Support and Hypotheses Development

Bandura (1977) sociological theories of changing behavior gave rise to the idea of self-efficacy. All of this relates to a teacher’s confidence in his or her capacity to handle the tasks, responsibilities, and challenges that come with his or her professional job (e.g., didactical tasks, managing discipline problems in the class, etc.) (Caprara et al., 2006). This notion is influenced by a number of elements, including personality features, particularly personality traits, which have sparked increased scholarly interest in the subject. Djigić et al. (2014) discovered that instructors with greater degrees of openness to experience and conscientiousness reported a stronger sense of efficacy, based on the five-factor model of personality. Self-efficacy is a critical process for behavioral change. It causes cognitive events that cause behavior to be initiated in order to attain a specific objective. This cognitive event, on the other hand, is impacted by mastery experience gained from earlier good performance (Bandura, 1982).

Situational variables, verbal persuasion, and physiological sensations are also sources of self-efficacy beliefs. Such an approach stresses the interplay of personal (psychological) characteristics, individual behavior, and environmental influences (Skaalvik and Skaalvik, 2007). With the complex structure of self-efficacy in mind and its relationship to a person’s view about his own efficacy in many realms of personal functioning or task completion, it is worthwhile to investigate the idea of teacher self-efficacy. Bandura created the measure of teacher self-efficacy among several other self-efficacy assessments in many areas (Bandura, 2006). Skaalvik and Skaalvik (2010) created the idea of teachers’ self-efficacy in light of the impact of self-efficacy on teacher efficacy. We assumed that teachers’ self-efficacy is the major contributor to creating brand equity at institutions, therefore, based on this theory, we proposed teachers’ self-efficacy as the independent variable of the current study influencing employee-based brand equity.

The resource-based theory has evolved through time to become a widely accepted research approach for establishing the link between efficiency and financial performance. The reorganization and incorporation of the resource create its uniqueness, allowing it to better adapt to the demands of a dynamic world (Barney, 1991) and, as a result, raise the chances of higher results (Song et al., 2005). Some researchers used the phrase “organizational unique competence” in 1957, and this idea was born (Song et al., 2005). Penrose proposed the “Theory of the Dynamic Growth of the Firm” in 1959, based on her economic theory, and defined Core Competency as an organization that obtains economic rents via the allocation and utilization of resources (Kor and Mahoney, 2004). Later, the resource-based theory was founded on this premise. According to resource-based theory research, the concept of human resources is indicated as innate organizational traits or as causal uncertainty, also known as the isolation mechanism (Lippman and Rumelt, 1982; Knott, 2003).

Nevertheless, why resources variety and uniqueness might boost the performance of the company, competitive edge, or organization performance requires greater clarity and investigation (Acedo et al., 2006). As per the above, the compatibility of the supply and demand of human capital between company and school would be represented in the college students’ employment rate when the basic and professional skills of academically trained student resources fulfill the industry’s need for student resources. All these things lead to academic psychological capital among students (Clarence et al., 2021), and academic PsyCap could lead to developing brand equity therefore, we utilized it as a mediator of the current study. The other mediators of the current study included student engagement and this variable was derived through flow theory (Shernoff et al., 2014). This stated that engaged students would be more helpful when getting user input through efficient teachers and would outflow the desired results.

### Teachers’ Self-Efficacy and Employee Based Brand Equity

The research of self-efficacy as well as its relationship to human behavior has expanded to include the efficacy of teachers, in particular. It has already been studied in terms of the impact on student achievement. Several studies have found a correlation between teacher efficacy and student accomplishment, which is the most important indicator of teacher effectiveness (Lee et al., 2013; Ababneh and Hackett, 2019; Madigan and Kim, 2021). Researchers have looked at the relationships between a teacher’s level of efficacy and his or her intention to embrace educational advancement, teachable moment presenting and questioning, power to control the level of stress, desire to stay in the field, and educational commitment, special education recommendation, and predictions of student success, in addition to student-related issues like an accomplishment, sense of self-efficacy, encouragement, and so on (Kim et al., 2017; Donohoo, 2018). In a larger sense, teacher self-efficacy is seen to be linked to certain other qualities including job involvement, perfection, and interpersonal skills.

Teacher efficacy, on the other hand, has been linked to teacher burnout (Motallebzadeh et al., 2014; Kim et al., 2017; Harrichand et al., 2021). Occupational stress has been related to overall self-efficacy, teachers’ self-efficacy, and even group self-efficacy (Tzioti et al., 2010). Researchers also looked at the link between occupational stress and the three forms of self-efficacy, as well as instructors’ evaluations of certain job values. The three categories of self-efficacy were shown to be adversely linked with the three characteristics of job burnout. Furthermore, Canrinus et al. (2012) looked at the relationship between significant measures of teachers’ aspect of professional identification (work-related commitment, work satisfaction, and motivation change). Stirin Tzur et al. (2016) discussed the debate concerning the direction of self-influence efficacy on achievement. They hypothesized that self-efficacy does have a beneficial influence on performance when the reward is high, but a negative effect when the reward is low. None of the prior studies evaluated the role of teachers’ self-efficacy on employee-based brand equity and provided us the opportunity to assess its impact on EBBE. Therefore we suggested the following hypothesis.

*H*_1_ *: Teachers’ self-efficacy has an association with EBBE*

### Teachers’ Self-Efficacy and Collective Self-Efficacy

Self-efficacy and collective efficacy are two distinct notions. Whereas self-efficacy refers to a person’s conviction in their capacity to complete a task, collective efficacy refers to the institution’s belief in its ability to do so (Bandura, 2001). Teachers’ self-efficacy views in the educational setting are based on personal performance judgments, while collective efficacy is based on societal perceptions of school staff capabilities as a whole (Thornberg et al., 2020). Bandura (1982) stated in his early investigations that the origins of self-and collective efficacy are the same, and also that collective efficacy is embedded in self-efficacy. Participants’ assessments of their group and the collective efficacy of respective organizations, particularly those in major management and leadership responsibilities, may be heavily impacted by their self-efficacy. The current study offered the following hypothesis based on the above findings.

*H*_2_ *: Teachers’ self-efficacy has an association with collective efficacy*

### Teachers Self-Efficacy and Academic PsyCap

Teachers’ self-efficacy is basically a component of academic psychological capital and no prior research has evaluated the impact of teachers’ self-efficacy on academic PsyCap in past. Teachers’ self-efficacy beliefs, of course, are not independent of other psychosocial factors that affect their productivity and commitment, including their competent ambitions, the acknowledgment and honor they believe they receive, and ultimately, the satisfaction they derive from their profession. Previous research has shown that a teacher’s self-efficacy attitudes have a significant effect on the overall performance and enthusiasm (Mikus and Teoh, 2021). Various researchers have focused on the psychological capital of teachers in different contexts indicating that teachers’ psychological capital could influence job satisfaction among school or college teachers (Aydin Sünbül and Aslan Gördesli, 2021), psychological capital promotes teachers’ wellbeing (Shen et al., 2014), and PsyCap and teachers’ effectiveness (Wang et al., 2014). This kind of relationship between academic PsyCap and teachers allowed us to develop a hypothesis to test the otherwise relationship between teachers’ self-efficacy and academic PsyCap.

*H*_3_ *: Teachers’ self-efficacy has an association with academic psychological capital*

### Teachers’ Self-Efficacy and Students Engagement

In comparison to the limited evidence that links teachers’ self-efficacy and responsibility to student engagement practices, substantial evidence based on self-determination theory suggests that students who feel backed in their need for independence and comforted by their teachers are much more involved in learning and gaining knowledge more. Student engagement describes the degree to which students participate in the instructional experience, and it is a necessary condition for learning and achievement. Student engagement takes several forms: Emotional involvement is a measure of a student’s enthusiasm for education activities (Flunger et al., 2019). Several other studies have also looked into the relationship between teachers’ self-efficacy and student’s engagement in learning (Wang and Fredricks, 2014; Shoulders and Krei, 2015; Lauermann and Berger, 2021). Therefore based on this analogy, we devised the following hypothesis.

*H*_4_ *: Teachers self-efficacy has an association with students’ engagement*

### Mediating Roles of Collective Self-Efficacy, Academic PsyCap, and Student Engagement

People’s shared confidence in their collective capacity to generate desired results is referred to as collective efficacy, which is based on social cognition theory (Chen, 2015). Collective effectiveness displays the ability of participants to act on one another’s behalf irrespective of pre-network links, highlighting the potential to mobilize social support in times of need. As a result, whether individuals would devote these resources to specific activities, such as catastrophe preparedness, is determined by their perceived collective efficacy. Reported collective efficacy has been linked to pro-environmental behavior, self-reported wellness, recovery from post-traumatic psychological stress, and catastrophe preparation in empirical investigations (Kawachi et al., 2008; Cagney et al., 2009; Heid et al., 2017; Babcicky and Seebauer, 2020). As discussed in the previous section, collective self-efficacy is affected by teachers’ self-efficacy and drawn from these studies, we assumed its mediating role between teachers’ self-efficacy and employee-based brand equity.

PsyCap is thought to be flexible and open to development (state-like), implying that it is not static and is more receptive to change in terms of positive resources and development via brief intervention. The influence of PsyCap has mostly been evaluated in terms of management satisfaction, productivity, and well-being, as well as at the organizational level among personnel. Research from such studies indicates that there is a positive relationship between these three dimensions (Georgiou and Nikolaou, 2019; Gomes da Costa et al., 2021). Surprisingly, research on the impact of PsyCap on university students’ well-being and academic performance has been scarce thus far. Some of the researchers looked at the relationship between PsyCap and students’ grades. The findings reveal that PsyCap is a favorable indicator of students’ grades, with greater PsyCap levels indicating better grades, and vice versa (Vanno et al., 2014). PsyCap’s involvement as a possible mediator in the relationship between academic stress and psychological symptoms (e.g., anxiety, somatic symptoms), physical ailments (e.g., migraine, queasy stomach), and life satisfaction have been investigated in a different way (Riolli et al., 2012).

Several scholars have analyzed the mediating role of students engagement previously (Siddiqi, 2018). Students’ engagement is long been seen as the most important educational result since it demonstrates that they are studying and performing well (Reeve and Tseng, 2011). According to scientists, student engagement seems to have a direct influence on academic success (Khoza, 2018). Students’ participation in literature has been described using a variety of categorizations. Student engagement, according to Kuh et al. (2008), is measured by how much performance and energy students devote to educational activities (Collaborating and Interacting). Students’ involvement, according to Fredricks et al. (2004) is a mix of several components such as emotional, social, and intellectual engagement. Some other researchers such as García-Martínez et al. (2021) utilized students’ engagement as mediators in their contexts. Though, no prior research evaluated the impact of collective self-efficacy, academic PsyCap, and student engagement on employee-based brand equity but all the supporting literature discussed above allowed us to develop the following hypothesis suggesting mediating roles of these variables.

*H*_5_ *: Collective self-efficacy mediates the relationship of teachers’ self-efficacy and EBBE*
*H*_6_ *: Academic PsyCap mediates the relationship of teachers’ self-efficacy and EBBE*
*H*_7_ *: Student engagement mediates the relationship of teachers’ self-efficacy and EBBE*

**FIGURE 1:**
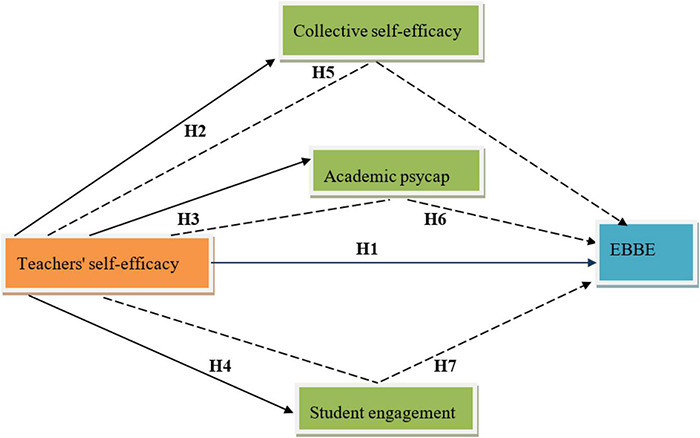
Conceptual model.

The following conceptual model (Figure 1) has been formed based on the above literature and hypotheses.

## Methodology

Considering the aim of this study, the data was collected from Chinese college students using the convenience sampling technique (Avotra et al., 2021). The population of Chinese college students has been targeted because they are the best stakeholder in the university and faculties there (Baig and Waheed, 2016). The convenience sampling technique has been recommended due to the pros it offers to the researcher; for example, it is cost-effective and the respondents are easily available. Furthermore, this technique allows the researcher to redo the data collection if any issue occurs at a later stage (Nawaz et al., 2020; Yingfei et al., 2021). However, in this study, the data was collected once making it a cross-sectional study. The study offers the causal frameworks measuring the effects of independent variables on the dependent variables, thus following a positivist philosophy of research supporting the deductive approach. The data collection had been administered by the researcher to minimize spurious results. The questionnaires were distributed to the potential respondents giving an orientation about the purpose of the study taking consent from the potential respondents if they are willing to participate. The anonymity of the respondents had been ensured by not giving an option to put their names on the questionnaire. The cover letter explained that the study is voluntary, data will be kept confidential, and data collected will be used for the analysis of the study only, thus meeting the criteria of ethical consideration of research. These steps helped to avoid bias and social desirability (Nawaz et al., 2020; Yingfei et al., 2021). A total of 400 questionnaires were distributed to the college students and 351 questionnaires were received. Participants were reached through personal visits and emails. After screening, 316 questionnaires were selected for analysis. Therefore, the usable questionnaires obtained from participants are 79%. Regardless of any social/professional influences, the data were collected on the free consent and willingness of the respondents. The authors compared with respect to late and early responses and no difference was found. The informed consent of the participants was implied through investigation achievement.

### Statistical Tool

The statistical tool used in this study for the analysis is Smart PLS 3.3.5 as it provides robust results and is user-friendly. This software helped the researchers to carry out the analysis using the partial least square structural equation modeling technique. Further, it treats small data with equal robustness as larger sample sizes (Nawaz et al., 2019; An et al., 2021). Smart PLS evaluates the results in two stages, i.e., measurement model and structural model stages. The first stage of the measurement model gives insights into the internal consistency and validity of the data obtained through questionnaires. The second stage, structural model measurement, gives the output for the hypotheses, formulated in the literature, for their acceptance or rejection based on the inbuilt statistical estimations considering *t*-test, *p*-values, etc.

### Measurement

The scales used in the present study have been adapted from past studies. The details for all scales have been given in the following.

#### Teacher Self-Efficacy

The shorter version teacher’s self-efficacy scale (five items) is adopted from Tschannen-Moran and Hoy (2001) this scale uses five points Likert scale to measure the teacher’s self-efficacy.

#### Student Engagement

Student engagement is measured on 5-point Likert (1 indicates I totally disagree and 5 indicates I totally agree) scale with five statements from Gunuc and Kuzu (2015). These five statements measure the class engagement and emotional engagement of college students.

#### Academic Psychological Capital

This scale is adopted from Luthans et al. (2012) with five items and this scale uses five responses they are Strongly Agree, Agree, Somewhat Agree, Somewhat Disagree, and Strongly Disagree.

#### Employee Based Brand Equity

The scale is based on questions adopted from the study by Mohan and Sequeira (2016). Ten items are used to measure different aspects of employee-based brand equity. A 5-point Likert scale is used 5 (Strongly Agree) and 1 (Strongly Disagree).

#### Collective Self-Efficacy

Collective self-efficacy has been defined as the group’s belief that it can perform the tasks and goals successfully. This scale is adopted from Little and Madigan (1997) and it has five items measured on a 9-point Likert scale one (Not at all confident) to nine (Completely confident).

### Demographic Analysis

The demographic profile of the respondents has been analyzed based on the two main characteristics of age and gender. The target population of the study was students who are generally characterized based on these two categories. Regarding age, the highest number of respondents was from the age group 18–25 making up 58.8% of the respondents. Regarding gender, male and female participation had been almost equal making 51.26% of male respondents and 48.73% of female respondents (see Table 1).

**TABLE 1:** Demographic analysis.

Demographic variable	Characteristics	Frequency	Percentage (%)
Age (years)	18–25	86	18.2
	26–30	145	30.7
	31–35	109	23.0
	36 and above	74	15.6
Gender	Male	429	90.7
	Female	44	9.2

## Data Analysis and Results

### Measurement Model

**FIGURE 2:**
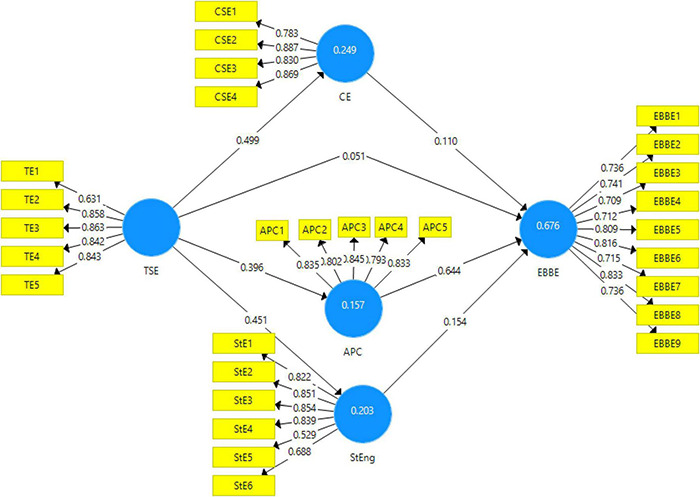
Output of measurement model.

The variance-based PLS-SEM is used in Smart-PLS in order to calculate the reliability of the measurements through Cronbach’s alpha, discriminant validity, convergent validity, and indicator reliability (Hair et al., 2014). These measures help is validate the measurement model of our framework (see Figure 2).

The measurement model has generated the results for the reliability and validity of the scales used in this study. Table 2 exhibits these results. These results have been estimated through factor loadings, Cronbach’s alpha, composite reliability, average variance extracted (AVE), and the variance inflation factor (VIF). According to different authors, for results to be valid, one of the measuring criteria is that the items show a factor loading of above 0.5 (Jordan and Spiess, 2019). The present study shows that all the items included in the study have shown factor loading of above 0.5 which shows the items are measuring the dimension of the variable that is expected to measure. Furthermore, the AVE values, according to the authors, are said to be more than 0.5 to show that items measure a variance more than the errors in that particular variable (Dash and Paul, 2021). AVEs for the present study have also been calculated to be above 0.5 indicating an adequate measure of variance. Furthermore the VIF, according to authors, has been said to show values less than 5 as it indicates the moderate correlation of the items with each other (Craney and Surles, 2007). Hence, in this study, the VIF values reported by the software are all below five, thus showing that the items of each variable are moderately correlated with each other.

**TABLE 2:** Model measurement.

Construct/Indicators	Loadings/Alpha	Composite reliability	AVE	VIF
**Academic psychological capital**	**α = 0.882**	**0.912**	**0.675**	
APC1	0.834			2.908
APC2	0.802			2.482
APC3	0.845			2.094
APC4	0.794			1.806
APC5	0.832			2.603
**Employee based brand equity**	**α = 0.906**	**0.922**	**0.570**	
EBBE1	0.766			2.218
EBBE2	0.776			3.010
EBBE3	0.740			2.459
EBBE4	0.714			1.661
EBBE5	0.782			2.923
EBBE6	0.791			3.232
EBBE7	0.684			2.108
EBBE8	0.808			3.590
EBBE9	0.725			1.928
**Student engagement**	**α = 0.859**	**0.897**	**0.598**	
SE1	0.822			2.538
SE2	0.851			2.697
SE3	0.854			2.598
SE4	0.840			2.563
SE5	0.728			1.799
SE5	0.688			2.087
**Collective self-efficacy**	**α = 0.864**	**0.908**	**0.711**	
CSE1	0.785			1.580
CSE2	0.887			2.827
CSE3	0.830			2.213
CSE4	0.868			2.352
**Teachers self-efficacy**	**α = 0.867**	**0.905**	**0.659**	
TSE1	0.652			1.323
TSE2	0.857			2.360
TSE3	0.858			2.465
TSE4	0.835			2.302
TSE5	0.837			2.272

*Bold indicates the relationship between variables.*

Moving to the internal consistency of the variables used in this study, the Cronbach’s alpha and composite reliability have been used to check the reliability of the variables used. According to past research, the cut-off values set for the acceptance of the criteria for a variable to be reliable are 0.7. Peterson and Kim (2013) showed that the statistics shown corresponding to these tests should be more than the said value. In this study, both the Cronbach’s alpha and composite reliability show statistics above 0.7; hence, indicating that the scales used for the present study will generate similar results when will be used in the future thus establishing the reliability of the measured constructs.

In addition to the aforementioned tests used (factor loading, AVE, and VIF) used for validating the scales, the Fornell and Larcker Criteria and hetero trait mono trait ratios have also been introduced in the present study to authenticate the results obtained by previous tests. The Fornell and Larcker Criteria indicate the correlation among the variables used in the study (Henseler et al., 2015). According to Fornell and Larcker (1981), the results are said to show valid correlations among the variables when the first value in each column is the highest than the rest of the beneath values. The present study results show for Fornell and Larcker Criteria have been reported in Table 3 and meet the acceptance criteria and the bold values reported in the table show that the variables used in this study are valid.

**TABLE 3:** Discriminant validity (Fornell and Larcker Criteria).

	APC	CE	EBBE	StEng	TSE
APC	**0.822**				
CE	0.462	**0.843**			
EBBE	0.480	0.562	**0.755**		
StEng	0.728	0.613	0.562	**0.773**	
TSE	0.400	0.500	0.570	0.453	**0.812**

*APC, academic psychological capital; CE, collective Efficacy; EBBE, employee based brand equity; StEng, student engagement; TSE, teachers self–efficacy. Bold indicates the relationship between variables.*

Similarly, another measure used to check the validity of the scale is the HTMT ratio (Henseler et al., 2015). The results of the test indicate that the scales used in this study to measure the concepts are valid. The acceptance of the HTMT ratio is based on the values generated for this test which should be less than 0.85. In the present study, the values obtained show the statistics of less than 0.85, hence, endorsing the validations proposed by the previous tests (see Table 4).

**TABLE 4:** Discriminant validity (HTMT ratio).

	APC	CE	EBBE	StEng	TSE
APC					
CE	0.502				
EBBE	0.502	0.610			
StEng	0.847	0.696	0.617		
TSE	0.438	0.577	0.885	0.525	

*APC, academic psychological capital; CE, collective Efficacy; EBBE, employee based brand equity; StEng, student engagement; TSE, teachers self-efficacy.*

Furthermore, the model fit of the study has been measured through *r*-square, *f*-square, and *q*-square values. *R*-square indicates how well does the data obtains fits in the regression line (Archer et al., 2021). The variable academic psychological capital shows that the data 16% fits the regression line. The highest fit has been obtained for the variable employee-based brand equity which shows a 40% fit. The following data fit is obtained for collective self-efficacy (25%) and the student’s engagement variable shows 20.5%. The *f*-square values indicated the strength of the relationship among the variables (Shukla, 2021). These are divided into three categories as small (*f*-square = 0.02), medium (*f*-square = 0.15), and high effect (*f*-square ≥ 0.35). For the present study, the independent variable teachers’ efficacy has shown moderate effect sizes with its corresponding dependent variables. The highest *f*-square value has been obtained for the relationship between teachers self-efficacy and collective efficacy (0.33) followed by the relationship between student engagement (0.25) and academic psychological capital (0.19). In addition, employee-based brand equity has shown a moderate relationship with collective self-efficacy (0.12), and weak relationship with student engagement (0.039), and a weak relationship with academic psychological capital (0.015). Furthermore, the *q*-square shows the predictive relevance (Hasan and Bao, 2020) and it is suggested to be greater than zero to show the relevance of the variables. In this study, all the variables have shown predictive relevance of more than zero (Academic psychological capital = 0.095, collective efficacy = 0.166, employee-based brand equity = 0.203, student engagement = 0.114, and teachers self-efficacy = 0.134).

### Structural Model

**FIGURE 3:**
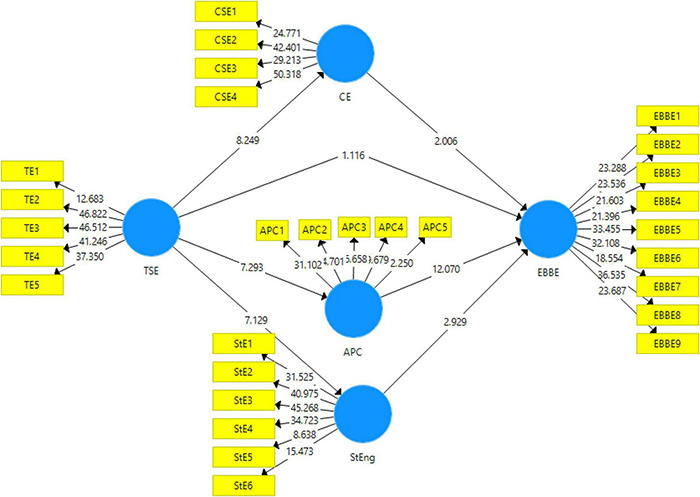
Output of structural model bootstrapping.

The software Smart PLS, in the structural model, uses bootstrapping technique to test if the data obtained support the hypotheses or not. In the present study, the statistics used to check the significance of the hypotheses are β values, *t*-statistics, and *p*-values. The acceptance criterion for t-statistic is that it should be more than 1.92, *p* value less than 0.05, VAF (variance accounted for) (Saricali et al., 2020). The data is run for bootstrapping at 5,000 subsamples at a 95% confidence interval (see Figure 3).

Table 5 reports the direct effects of the study. The first direct effect is between the teachers self-efficacy and the employee based brand equity indicating insignificant results with *t* = 1.16 and *p* > 0.05. The second, third, and fourth hypotheses of the study indicate the direct effects of teachers self-efficacy on collective self-efficacy (β = 0.499, *t*-value = 8.24, *p* = 0), academic psychological capital (β = 0.396, *t*-value = 7.29, *p* = 0) and students engagement (β = 0.451, *t*-value = 7.12, *p* = 0) have been accepted indicating strong direct effects.

**TABLE 5:** Direct effects.

Hypothesis	Beta	*SD*	*T* statistics	*P*-values
**H_1_:** Teacher’s self-efficacy → employee based brand equity	0.051	0.046	1.116	0.265
**H_2_**:Teacher’s self-efficacy **→** collective self-efficacy	0.499	0.060	8.249	0.000
**H_3_**:Teacher’s self-efficacy **→** academic pschyCap	0.396	0.054	7.293	0.000
**H_4_**:Teacher’s self-efficacy **→** student’s engagement	0.451	0.063	7.129	0.000

The indirect effects of the study indicating the mediation effects between the relationship of teachers self-efficacy and EBBE have been reported in Table 6. These hypotheses have been accepted/rejected based on the values of β, the *t*-statistic, variance accounted for, and *p*-value. There have been mentioned three interpretations of VAF weak (<20%), moderate (20% < VAF < 80%), and strong (>80%). The table shows that collective self-efficacy (β = 0.341, *t*-value = 6.22, *p* = 0) and student engagement (β = 0.172, *t*-value = 3.57, *p* = 0) have moderately mediated the relationship between teachers self-efficacy and EBBE. On the other hand, academic psychological capital has strongly mediated the relationship between teachers self-efficacy and EBBE with β = 0.113, *t*-value = 2.27, *p* = 024).

**TABLE 6:** Indirect effects.

Hypothesis	Beta	VAF (%)	*SD*	*T* statistics	*P*-values
**H_5_:** Teacher’s self-efficacy → collective self-efficacy→ employee based brand equity	0.341	51.83%	0.055	6.227	0.000
**H_6_:** Teacher’s self-efficacy → academic PsyCap→ Employee based brand equity	0.172	83.33%	0.048	3.573	0.000
**H_7_:** Teacher’s self-efficacy → student engagement→ employee based brand equity	0.113	57.61%	0.050	2.270	0.024

## Discussion

This research is a novel approach to determining employee based brand equity in educational institutes. University or college brand equity is slightly a newer concept in which educational institute develops as a brand. The faculty and other stakeholders of the educational institute develop brand equity in one or another way. College and universities’ management is increasingly realizing that having a good knowledge of brand equity may help them discover the characteristics that bring value to the brands and position them well in the market (Partenie, 2019; Dajani et al., 2021). Moreover, there is hardly any scientific publication on management comprehension of college brand equity, which is fascinating. In particular, when it comes to brand management in emerging markets, less is known about the notion (Pinar et al., 2014). Therefore, this study was carried out to evaluate the role of teachers in terms of their efficacy in a contribution to brand equity. The specific dimension of brand equity, i.e., employee-based brand equity was the main focus of this research. Different direct and indirect relationships of efficacy and EBBE were analyzed in this research based on the responses from college students of China. The direct relationship of teachers’ self-efficacy in building employee based brand equity was the main focus of this research.

The results provided significant insights into the role of teachers’ self-efficacy in higher educational institutes such as colleges and universities. Developing college and universities into the brand and promoting employee-based brand equity in these institutes got new support from current research. Multiple studies have been conducted on the branding and marketing of institutions of higher learning over the years. The focus of the preliminary studies appears to be focused on marketing and advertising aspects only (Argenti, 2000; Fazli-Salehi et al., 2019). Academic program repute, tuition, pamphlets, relationships between teachers, students, employees, and electronic media, as well as publicity, were all key variables for students when choosing a business school (Ryńca and Ziaeian, 2021). Some other research focused on the role of interactions between teachers, students, staff, and the community in university marketing. Furthermore, amenities were discovered to be crucial for institution branding (Girard and Pinar, 2021). Previously the only focus was on evaluating the effectiveness of teaching but by that time research on self-efficacy as well as its relationship to human behavior expanded to include the efficacy of teachers, in particular.

It was already studied in terms of the impact on student achievement. Several studies found a correlation between teacher efficacy and student accomplishment, which was the most important indicator of teacher effectiveness (Lee et al., 2013; Ababneh and Hackett, 2019; Madigan and Kim, 2021). The other contributors to current research aided in developing employee-based brand equity. The direct relationship of teachers’ self-efficacy with collective efficacy provided a strong indicator that if teachers are efficient than it does have an impact on others collectively. Teachers’ self-efficacy views in the educational setting were based on personal performance evaluations, while collective efficacy was based on societal perceptions of school staff capabilities as a whole (Thornberg et al., 2020). Therefore, results proved that teachers’ self-efficacy would be useful for developing collective efficacy. The impact of teachers’ self-efficacy on academic psychological capital was also addressed in this research which indicated that if teachers were efficient then it helped in developing psychological capital academically. If teachers had no self-efficacy then it would not have helped in developing academic PsyCap.

Similar studies have also been carried out in past in different perspectives in which psychological capital induced self-efficacy among teachers of higher education (Aydin Sünbül and Aslan Gördesli, 2021). Various researchers focused on psychological capital of teachers in different contexts indicating that teachers’ psychological capital could influence job satisfaction among school or college teachers (Aydin Sünbül and Aslan Gördesli, 2021), psychological capital promoting teachers’ wellbeing (Shen et al., 2014), and PsyCap and teachers’ effectiveness (Wang et al., 2014). It was also evident from the results that teachers’ self-efficacy had a significant contribution toward engaging the students which produced useful outcomes among students. Several other studies also looked in to the relationship between teachers’ self-efficacy and students engagement in learning (Wang and Fredricks, 2014; Shoulders and Krei, 2015; Lauermann and Berger, 2021). The indirect effects of three mediators were also studied in current research. All three mediators provided a handful of help in directing the relationship between teachers’ self-efficacy with employee-based brand equity.

Previously, no research was carried out in a similar fashion to this research in which collective self-efficacy could mediate the relationship of EBBE. Though, collective efficacy showed that it was linked to pro-environmental behavior, self-reported wellness, recovery from post-traumatic psychological stress, and catastrophe preparation in empirical investigations (Kawachi et al., 2008; Cagney et al., 2009; Heid et al., 2017; Babcicky and Seebauer, 2020). Similarly, academic PsyCap and student engagement were also not utilized in a similar fashion to this research but both of these significantly mediated the relationship of teachers’ self-efficacy with developing EBBE. While, PsyCap’s involvement as a possible mediator in the relationship between academic stress and psychological symptoms (e.g., anxiety, somatic symptoms), physical ailments (e.g., migraine, queasy stomach), and life satisfaction were investigated from different perspectives (Riolli et al., 2012). Likewise, several scholars analyzed the mediating role of students’ engagement from different perspectives such as reported by Siddiqi (2018).

### Practical Implications

The present study offers certain practical implications for universities and other organizations. For example, first of all, universities should conduct frequent seminars and workshops that actively involve the faculties and motivate them toward their jobs by strengthening their commitment to academics. Second, universities administration is increasingly realizing that having a good understanding of brand equity may help them in practicing such activities that bring value to their universities and position them better in the overall market. Thirdly, the faculties and universities should design such teaching methodologies that include team assignments that encourage the students to actively engage and learn how to participate and perform in teams, hence motivating teachers to give their best toward better performance.

### Theoretical Contribution

The present study strongly contributes by offering a scientific study to understand comprehensively the role of teachers self-efficacy in university brand equity. Particularly the brand management of universities in the markets is still an emerging concept. The present study has availed the opportunity to measure these relationships. In this regard, first of all, the present study has established that teacher self-efficacy contributes to the brand equity of universities indicating the mediation of academic psychological capital being the most important factor followed by student engagement and collective self-efficacy. Furthermore, it has also been established that teachers self-efficacy is an important contributing factor in collective self-efficacy followed by academic psychological contract and student engagement.

### Limitations and Future Recommendations

Along with the theoretical and practical importance of the study, there are some limitations as well that should be taken care of in future studies. The present study has used a convenience sampling technique which has its own drawbacks. Therefore, future studies are encouraged to conduct the study in a similar fashion to probability sampling to avoid any biases in results and offer more generalizable results. Secondly, teachers self-efficacy could not find any effect on the employ based brand equity being the major hypothesis of the study. Therefore, in future it should be tested in different settings to understand if this direct relationship exists between these two variables. Thirdly, moderation should be introduced in the framework offering more rigorous and robust results. The potential moderating variables could be organizational support, leadership, and also ethics will be an interesting moderating variables to check if the effect of teachers’ self-efficacy on student engagement is moderated. Furthermore, it will be interesting to add other mediating variables in the study like psychological contract breach, workplace incivility, or work stress.

## Conclusion

The teachers of higher education tend to associate with their institutions having higher self-efficacy toward their responsibilities hence giving students the confidence to participate in the class activities. The present study has examined the effect of teachers self-efficacy on the employee-based brand equity which could not find significance in this relationship. Moreover, teachers self-efficacy found significant effects on collective self-efficacy and academic psychological capital indicating that teachers involved in teaching activities create the feeling among students of having some academic achievement. Furthermore, teachers self-efficacy also found significant effect on student engagement showing that if teachers are self-motivated for doing their job makes students engage in their academic activities even more. Regarding the mediating effects of the study, collective self-efficacy, academic psychological capital, and student engagement were found to have a significant role in the relationship between teachers’ elf efficacy and employee based brand equity indicating how academic achievements and engagements of the students make teachers associate themselves with their institutions.

## Data Availability Statement

The original contributions presented in the study are included in the article/supplementary material, further inquiries can be directed to the corresponding author/s.

## Ethics Statement

The studies involving human participants were reviewed and approved by X’ian University of Architecture and Technology, China. The patients/participants provided their written informed consent to participate in this study. The study was conducted in accordance with the Declaration of Helsinki.

## Author Contributions

The author confirms being the sole contributor of this work and has approved it for publication.

## Conflict of Interest

The author declares that the research was conducted in the absence of any commercial or financial relationships that could be construed as a potential conflict of interest.

## Publisher’s Note

All claims expressed in this article are solely those of the authors and do not necessarily represent those of their affiliated organizations, or those of the publisher, the editors and the reviewers. Any product that may be evaluated in this article, or claim that may be made by its manufacturer, is not guaranteed or endorsed by the publisher.
